# Modeling and Performance Analysis of Uplink Laser Transmission Across Sea Surfaces: A Channel Characterization Study

**DOI:** 10.3390/s25041239

**Published:** 2025-02-18

**Authors:** Hong Gao, Tinglu Zhang, Ruiman Yuan, Lianbo Hu, Shuguo Chen

**Affiliations:** 1 College of Marine Technology, Faculty of Information Science and Engineering, Ocean University of China, Qingdao 266100, China; gaohong@stu.ouc.edu.cn (H.G.); m17854296271@163.com (R.Y.); hulb@ouc.edu.cn (L.H.); chenshuguo@ouc.edu.cn (S.C.); 2 Laboratory for Regional Oceanography and Numerical Modeling, Qingdao Marine Science and Technology Center, Qingdao 266237, China

**Keywords:** laser transmission, wave spectrum, spatiotemporal surface topography, Monte Carlo simulation, ray tracing

## Abstract

Variable marine environmental conditions, particularly at the sea surface, present considerable challenges to cross-media laser transmission. This study simulates uplink laser transmission through a seawater–sea surface–air channel via ray tracing and Monte Carlo methods, with an emphasis on the impacts of the sea surface channel. A spatial model of the sea surface is introduced, which uses a wave spectrum and fast Fourier transform technology, and the results are compared against those of a classical statistical model. The validity and applicability of six representative wind wave spectra are assessed for their effectiveness in characterizing the optical sea surface. Among these spectra, the Elfouhaily spectrum, which is refined for low-wind conditions, can most accurately represent the optical properties of the sea surface. The simulations reveal that the spatial model captures power fluctuations due to dynamic sea surface changes. At shorter underwater transmission distances, the spatial model may induce considerable drift, thereby degrading power estimates, where the difference is about 0.9 dB compared with the statistical model. Deeper underwater transmissions can mitigate beam distortions, resulting in a decrease in normalized peak power from −114 dB to −157 dB. Additionally, the laser centroid distribution tends to be elliptical because of the distribution of the sea surface azimuth. These findings underscore the importance of incorporating spatiotemporal dynamics in modeling sea surfaces and provide insights for optimizing underwater air laser transmission links in complex marine environments.

## 1. Introduction

The marine environment presents substantial challenges for cross-media laser transmission because of its dynamic nature [1,2]. Specifically, laser transmission at the sea–air interface is considerably affected by varying sea surface conditions [3]. The wind fields over the sea surface, along with temporal and spatial variations, alter the surface roughness, disrupting the path and integrity of laser beams [4,5,6]. This distortion and drift of laser signals hinder the effectiveness of technologies such as Light Detection and Ranging (LIDAR) and wireless optical communication (WOC) in the ocean [2,3,4,7]. Thus, it is imperative to accurately represent the morphologies and optical properties of sea surfaces and account for the effects of these disturbances on laser transmission. The application of an accurate sea surface model can considerably improve the design and functionality of ocean engineering and remote sensing applications [1,3,4,5,6,8,9,10,11]. This improvement is crucial for ensuring the reliability and efficiency of these systems, especially water–air laser transmission systems, in complex marine environments.

Initially, the sea surface model was simplified to assume a flat and static sea surface to streamline the setup of laser transmission links between underwater and satellite terminals and to facilitate power budget calculations [11,12]. However, a series of controlled laboratory tank experiments has clearly shown that alterations in the water surface and its slopes considerably influence the performance of laser transmission [4,5,6,10,13,14,15]. Even minor wave slope changes can deflect the laser beam, causing fluctuations in the received optical power, particularly in uplink transmissions [10]. In practical laser transmission systems, the varying sea–air interface can randomly alter the laser trajectory, intermittently disrupting the transmission channel. This variability compromises the reliability of the channel and causes the connection to switch intermittently between active and inactive states [13,14,15]. Hence, the sea surface model must accurately reflect the dynamic and temporal variations in surface slope and topography to improve the accuracy and performance of channel simulations.

Currently, the most widely used sea surface model is the Cox–Munk empirical model [16], which is used to simulate the statistical properties of ocean surface slopes for optical calculations [17,18,19,20,21,22,23]. Although this model accurately represents wave slopes across the full range of spatial scales, it overlooks the temporal and spatial dynamics of sea surface topographies, failing to display variations in received power [11,24]. In contrast, the wave spectrum-based model can effectively integrate the spatiotemporal characteristics of the sea surface topography and its slopes. To date, various wave spectral models have been utilized in cross-media laser transmission studies. For example, Zhang et al. [2], employing the Neumann spectrum, explored the dynamic interaction between underwater nodes and wave action, focusing on alignment deviations. Angara et al. [11] studied the influence of wind-driven waves through the Elfouhaily spectrum and bubbles on WOC systems under varying sea conditions. Alharbi et al. [14] employed Pierson–Moskowitz and JONSWAP (Joint North Sea Wave Project) spectra to assess the slope of water surfaces and refraction angles under varying environmental conditions. Similarly, Zhou et al. [25] examined the optical field distribution of an upward-transmitted laser across the water–air interface via the Pierson–Moskowitz spectrum. These studies underscore the utility of wave spectral models in enhancing the knowledge of laser interactions with complex oceanic surfaces. However, these methods are limited to examining one or two specific models without a performance assessment in simulating the optical properties of sea surfaces. Selecting an optimal spectrum according to the experimental objectives remains an important prerequisite, especially in comparison with the classical statistical model.

In this study, the uplink process of laser transmission from seawater to air channels is modeled based on the ray tracing technique and Monte Carlo simulation. To simulate sea surfaces efficiently, this study first evaluates the spectral characteristics of six typical wave spectra. To enhance the comparison of optical properties among different wave spectra, the often-overlooked truncation error of the Elfouhaily spectrum at low wind speeds is analyzed and refined. Subsequently, the mean square slope (MSS) of the simulated sea surface is computed to aid in selecting the most suitable spectrum for accurately modeling sea surface dynamics and laser channel transmissions. Afterward, the effects of different wind speeds and underwater depths on the beam power reaching the ideal and optimal receiving plane are investigated. In particular, this study introduces the time evolution property alongside spatial averaging statistics, enabling a comprehensive comparison of the laser transmission characteristics through both statistical and spatial sea surfaces.

The remainder of this paper is structured as follows: In Section 2, the sea surface channel model is presented, including the classical statistical sea surface and the wave spectrum simulation sea surface, with a focus on refinement of the truncated characteristics of the Elfouhaily spectrum at low wind speeds. In Section 3, the uplink seawater–sea surface–air channel is described, incorporating simplifications for the optical properties of the water and air columns. In Section 4, numerical results, including power spatial distributions, and beam offsets, are analyzed. Finally, this paper concludes with a summary in Section 5.

## 2. Sea Surface Model

A primary challenge in marine cross-media laser transmissions is the dynamic features of sea surfaces [2,10]. Accurately understanding the impact of wave-induced disturbances on beam propagation is crucial for performing laser experiments in marine environments. In this section, sea surface channel models primarily utilize the Cox–Munk model wind-generated slope model (hereafter, the statistical model) and the simulated spatial model of sea surfaces (hereafter, the spatial model), which is implemented on the basis of a wave spectrum and the fast Fourier transform (FFT) technique.

### 2.1. Statistical Model

Results from a field experiment and in situ measurement show that the probability distribution function (PDF) of sea surface slopes can be approximated by a Gaussian distribution [16,26,27,28]:

(1) $$p\left(\theta \right)=\frac{2}{\pi \sigma^{2}}\exp\left[-\frac{\tan^{2}\theta }{\sigma^{2}}\right]\tan\theta \sec^{2}\theta$$

where θ is the isotropic surface slope, and $\sigma^{2}$ is the total MSS related to the wind speed U.

The cumulative distribution function (CDF) for θ is

(2) $$P\left(\theta \right)=1-\exp\left[-\frac{\tan^{2}\theta }{\sigma^{2}}\right].$$

Therefore, the polar angle of the normal to a wave facet can be written as tanθ = $\sqrt{-2\sigma^{2}ln(\eta )}$, and the isotropic azimuthal angle is φ = 2πη, where η is uniformly distributed between 0 and 1. The normal vector $\vec{n}$ with respect to the normal to the horizontal is a combination of the wave facet slope θ and direction φ [17,22,23].

The wind field above the sea surface influences the distribution of sea surface slopes by impacting the $\sigma^{2}$ values. Cox and Munk proposed a linear relationship between the MSS and wind speed for the open ocean based on aerial photographs [16]:

(3) $$\sigma^{2}=0.003+5.12\times 10-3U\pm 0.004$$

Furthermore, Hu et al. obtained a nonlinear relationship between the MSS and U by comparing sea surface backscatter data from Cloud-Aerosol Lidar and Infrared Pathfinder Satellite Observations (CALIPSO) with matched-up Advanced Microwave Scanning Radiometer for EOS (AMSR-E) wind speed measurements [29]:

(4) $$\sigma^{2}=\left\{\begin{array}{cc} 0.0146\sqrt{U}\|\| & U<7\;m\;s^{-1}\\ 0.003+0.00512U\|\| & 7\;m\;s^{-1}⩽U<13.3\;m\;s^{-1}\\ 0.138{log}_{10}U-0.084\|\| & U⩾13.3\;m\;s^{-1} \end{array}\right.$$

For wind speeds from 7 m s^−1^ to 13.3 m s^−1^, Equation (4) directly follows the formula of Equation (3), whereas a bias is observed at other wind speeds. This discrepancy is due to the sensitivity of the laser to finer capillary wave structures, resulting in higher values from Equation (4) at wind speeds below 7 m s^−1^ than those from Equation (3) [29]. Even though Equation (3) is commonly employed [17,18,19,20,21,22], Equation (4), derived from LIDAR data, may offer a suitable framework for analyzing laser transmission characteristics [23,27,28,29].

### 2.2. Spatial Model

The dynamic spatial and temporal properties of sea waves are of greater interest [30] than the statistical model is because of their potential to enhance communication performance [10]. Typically, the sea surface can be considered a summation of an infinite number of discrete sinusoidal wave components [26]. To realistically simulate waves, a reliable wave spectrum is required [31], which is also fundamental for accurately predicting laser beam interactions with the sea surface.

#### 2.2.1. Sea Spectra

Several previous studies have simulated the impact of sea surfaces on water–air laser transmission links, primarily concentrating on typical gravity wave spectra [2,14,25]. However, existing studies have indicated that wave height is predominantly influenced by gravity wave dynamics, whereas the slope of the sea surface is affected by short gravity waves and capillary waves [24,26,32]. When wave spectra are used to model the spatial characteristics of the sea surface, ensuring that the spectra exhibit robust performance across all wavenumbers is essential.

To conduct a systematic evaluation of diverse wave spectra for the application of laser transmission channel simulation, three representative composite spectra containing high-frequency wavenumbers were also selected, in addition to the typical gravity wave spectra. Table 1 presents detailed descriptions of the typical candidate spectra utilized in this comparative analysis.

Neumann [33] pioneered the empirical relationship between wave height and period assisted by apparent observations. Neumann first formulated a wave spectrum that correlated wind speed variations to the functional form of the long gravity wave spectrum. Pierson and Moskowitz [34] and Hasselmann et al. [35], drawing on the similarity hypothesis from Kitaigorodskii, meticulously processed and analyzed spectral data from the North Atlantic and North Sea westward from Sylt, respectively. Their efforts resulted in one-dimensional spectra that are highly effective and robust over time in characterizing gravity waves in fully developed sea waves [26,36,39].

Remote sensing technologies, through advanced observation equipment such as radar, altimeters, and LIDAR, offer new insights into the complex structures of waves. These technologies aid in detailing high-frequency wave patterns and improve the understanding of sea surface roughness [40]. The Apel [36], Elfouhaily [37], and Hwang [38] spectra are comprehensive wave spectrum models across full wavenumbers, utilizing extensive field and water tank data and citing radar backscatter, optical observations, and hurricane observation experiments, respectively.

The simulation of the sea surface employs the directional spectrum function:

(5) $$\Psi \left(k,\theta \right)=\frac{1}{k}S\left(k\right)D\left(k,\theta \right)$$

where S(k) is the omnidirectional spectrum and D(k,θ) is the spreading function [36]:

(6) $$D(k,\theta )=C_{s}{cos}^{2s_{p}}(\theta /2)$$

Here, $s_{p}$ is the wave propagation parameter, which is associated with frequency k, wind speed U, and wave age $\Omega_{c}$, and $C_{s}$ is the normalization parameter.

On the basis of the above equations, the variance of the zero-mean wave height and the total MSS can be calculated as follows [24]:

(7) $$\begin{array}{ll} \left\langle h^{2}\right\rangle & =\int_{0}^{\infty }\;\int_{0}^{2\pi }\;\Psi \left(k,\theta \right)k\;dkd\theta \\ & =\int_{0}^{\infty }\;\int_{0}^{2\pi }\;S\left(k\right)D\left(k,\varphi \right)\;dkd\theta \\ & =\int_{0}^{\infty }\;S\left(k\right)\;dk \end{array}$$

and

(8) $$\begin{array}{ll} \sigma^{2} & =\int_{0}^{\infty }\;\int_{0}^{2\pi }\;k^{2}\left({sin}^{2}\varphi +{cos}^{2}\varphi \right)\Psi \left(k,\varphi \right)k\;dkd\varphi \\ & =\int_{0}^{\infty }\;\int_{0}^{2\pi }\;k^{2}\Psi \left(k,\varphi \right)k\;dkd\varphi \\ & =\int_{0}^{\infty }\;k^{2}S\left(k\right)\;dk \end{array}$$

where $\left\langle h^{2}\right\rangle$ denotes the expectation of sea surface elevation; hence, the significant wave height can be expressed as $H_{s}$ = 4$\sqrt{\left\langle h^{2}\right\rangle }$. Furthermore, $S\left(k\right)$ is also called the elevation spectrum, which is provided by different wave spectral models, and $k^{2}S\left(k\right)$ is the slope spectrum.

**Figure 1:**
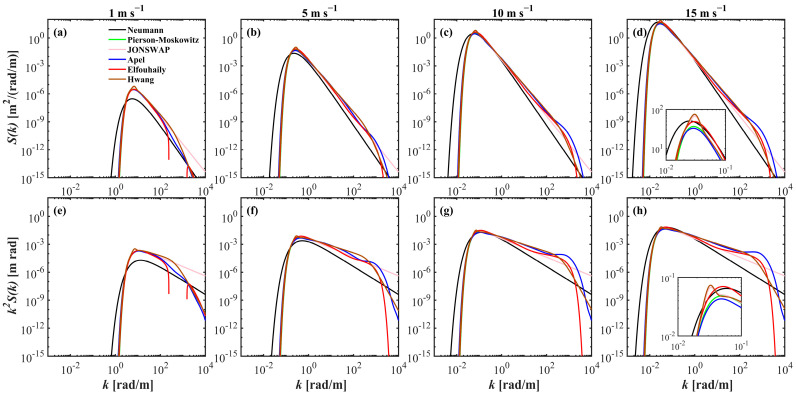
Comparison of elevation spectrum (**a**–**d**) and slope spectrum (**e**–**h**) across six different wave spectra for wind speed of (left to right) 1, 5, 10 and 15 m s^−1^, respectively. Each spectrum is represented by a unique color. (**a**,**e**) reveal truncations due to negative values in the Elfouhaily spectrum (red solid). The subplot examples in (**d**,**h**) feature detailed zoom-in on the wavenumber range from 0.001 to 0.1 rad m^−1^, enhancing the visibility of spectral nuances.

Figure 1a–d,f–h show the gradual shift in the elevation and slope spectra of the waves from high-frequency capillary waves to low-frequency gravity waves with increasing wind speed. The boundary between gravity and capillary waves is approximately 363 rad m^−1^. In the gravity wave range, the spectral shapes of the different wave spectra are almost identical, except for the Neumann spectrum, where the spectral slopes are −3 and −3.5, respectively. The differences among wave spectra become pronounced at higher frequencies, which represent the smallest gravity and capillary waves. Despite their minimal contribution to overall wave height variance, their slopes may vary greatly [24].

#### 2.2.2. Refinement of the Elfouhaily Spectrum

**Figure 2:**
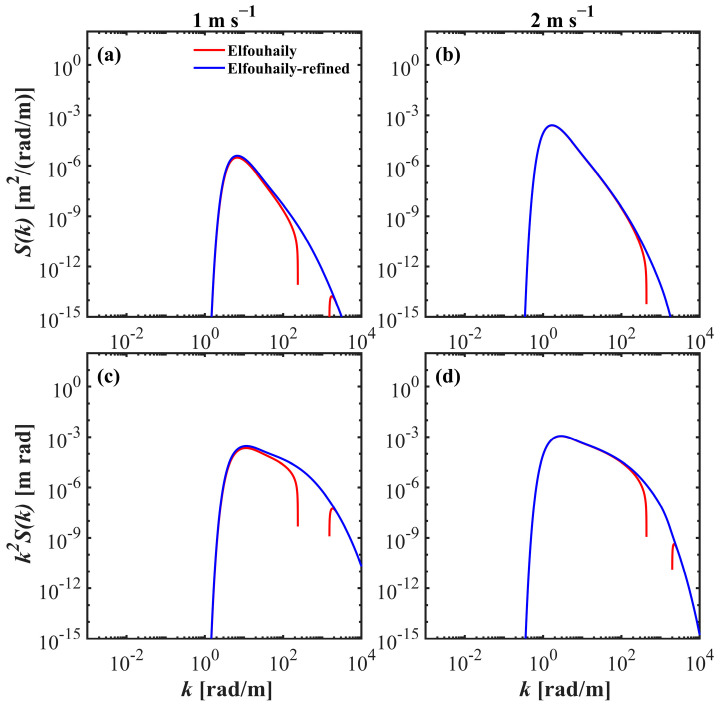
The Elfouhaily elevation spectrum (**a**,**b**) and slope spectrum (**c**,**d**) for wind speed of (left to right) 1 and 2 m s^−1^, respectively. The red line represents the values derived from the original spectrum, where the missing part is negative, and the blue line shows the refined results.

To explicate the spectral truncation depicted in Figure 1a,e, the process of modeling the Elfouhaily spectrum is reviewed. Elfouhaily et al. [37] introduced the generalized Phillips–Kitaigorodskii equilibrium range parameter $\alpha_{m}$ to describe the short-wave curvature spectrum. A two-regime logarithmic law was fitted on the basis of field experimental data as (Ref. [37], Equation (44))

(9) $$\alpha_{m}=\left\{\begin{array}{ll} 0.01[1+ln(\frac{U_{*}}{c_{m}})]\|\| & ifU_{*}⩽c_{m}\\ 0.01[1+3ln(\frac{U_{*}}{c_{m}})]\|\| & ifU_{*}>c_{m} \end{array}\right.$$

where $U_{*}$ is the friction velocity, which is approximately equal to 0.0378 U; $c_{m}$ can be considered a constant for 0.23 m s^−1^; and $\alpha_{m}$ is also called the saturation level of the secondary peak. It is important to note that due to the fitting data used by the Elfouhaily spectrum at low wind speeds, the measurements may be close to the noise level of the instrument, which may affect the fitting accuracy and lead to bias or failure at low wind speeds [37,41]. Thereafter, a threshold friction wind speed of approximately 0.085 m s^−1^, equivalent to a wind speed of approximately 2.25 m s^−1^, can be calculated for the Elfouhaily spectrum. If the wind speed is less than the threshold condition, the calculation of the spectral energy from the gravity waves to capillary ranges would take on negative values, resulting in the failure of the spectral bands (as shown by the red line in Figure 2). Therefore, it is necessary to combine a variety of observation instruments for comprehensive comparison to improve the reliability and consistency of data.

Similarly, Apel [36] also modeled a saturation exponential function from radar data, which is particularly effective for fitting low-wind-speed conditions, as (Ref. [36], Equation (15))

(10) $$\alpha_{m}=exp\left\{\left[-4.95+3.45\left(1-e^{\frac{-U}{U_{\eta }}}\right)\right]ln10\right\}$$

where $U_{\eta }$ is the saturation speed, which is 4.7 m s^−1^, and a secondary peak is assumed at 750 rad m^−1^.

Equation (10) can fit the saturation parameter accurately, whereas Equation (9) aligns closely with the experimental data at medium to high wind speeds [36,37,41,42]. Thus, in this study, the above two equations are combined to compensate for the instability of the Elfouhaily spectrum under low-wind-speed conditions.

In this instance, the refined Elfouhaily spectrum applies Equation (10) for wind speeds below 2.25 m s^−1^ and Equation (9) for all other conditions. The blue lines in Figure 2 illustrate examples of the results. This implementation enhances and broadens the detail regarding wind saturation effects and provides a reference for the spectral integrity of the Elfouhaily spectrum at low wind speeds.

#### 2.2.3. Sea Surface Simulations and Statistical Properties

The above results suggest that all the candidate wave spectra maintain stable performance across variable wind speeds and wavenumbers, allowing for robust calculation in the simulation of spatial surfaces. After specific environmental parameters, primarily wind field data, are prescribed, combining a wave spectrum with FFT techniques facilitates the efficient simulation of virtual realistic sea surfaces (publicly available from Refs. [24,43]). The simulated sea surface parameters are presented in Table 2. The wave length L is set to be at least twice the characteristic wavelength to ensure the simulation captures the full wavenumber spectrum. And the grid number N is chosen based on computational efficiency and empirical considerations. Notably, to compensate for the lack of sea surface information due to the discrete grid size used during the simulation process [44], Mobley suggested a spectral enhancement sampling method (Ref. [24], Equation (9)), which can effectively ensure that the simulated sea surface finite difference sampling results are consistent with the theoretical calculations. The simulation can capture the dynamics of waves more fully at temporal and spatial scales.

**Figure 3:**
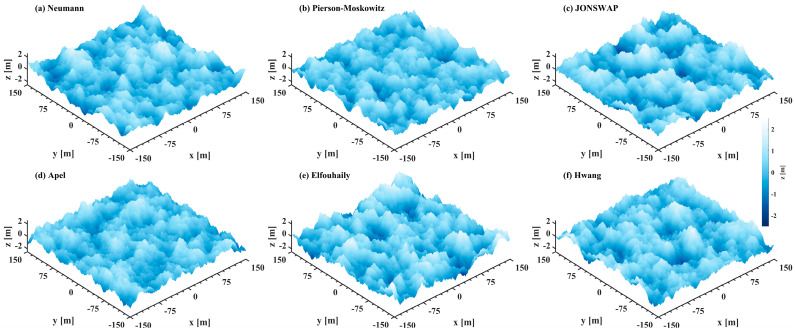
Simulated sea surface topographies across different sea spectra for a wind speed of 10 m s^−1^. All topographies represent wind-blown sea surfaces at full development.

Discrete, 2D random elevation grids are generated, as illustrated in Figure 3, which shows examples of simulated spatial wind-blown surfaces for a wind speed of 10 m s^−1^. The topographies of the sea surface obtained from different wave spectra are visually quite comparable, with wave heights distributed within ±2.5 m. Regarding the fine surface characteristics, the simulated sea surface displays greater roughness with the composite spectrum (Figure 3d–f) than with the typical gravity spectrum (Figure 3a–c).

When spatial models are applied, the key statistical properties of sea surfaces are elevation and slope [8]. The elevation provides insights into the physical features of topographies and the spatial distribution of slopes. In contrast, the slope characterizes the optical properties of surfaces and considerably influences the interaction of the beam with surfaces. Under these circumstances, finite differences must be applied to calculate the elevation and total MSS for each spatial surface. Therefore, Equations (7) and (8) can be reformulated accordingly as

(11) $$\begin{array}{ll} \left\langle h^{2}\right\rangle & =\sum z^{2}\\ & =\frac{1}{N_{x}N_{y}}\sum_{i,j}^{N_{x},N_{y}}\;{z(x_{i,j\;},{\;y}_{i,j})}^{2} \end{array}$$

and

(12) $$\begin{array}{c} \sigma^{2}=\sum [(\frac{\Delta z}{\Delta x}{)}^{2}+(\frac{\Delta z}{\Delta y}{)}^{2}]\\ =\frac{1}{\left(N_{x}-1\right)N_{y}}\sum_{i,j}^{N_{x},N_{y}}\;{\left[\frac{z\left(x_{i+1,j\;},{\;y}_{i,j}\right)-z\left(x_{i,j\;},{\;y}_{i,j}\right)}{x_{i+1,j}-x_{i,j}}\right]}^{2}\\ +\frac{1}{N_{x}(N_{y}-1)}\sum_{i,j}^{N_{x},N_{y}}\;{\left[\frac{z(x_{i,j\;},{\;y}_{i,j+1})-z(x_{i,j\;},{\;y}_{i,j})}{y_{i,j+1}-y_{i,j}}\right]}^{2} \end{array}$$

where $z(x_{i,j\;},{\;y}_{i,j})$ is the sea surface elevation at spatial coordinates $\left(x_{i,j\;},{\;y}_{i,j}\right)$, and $N_{x}$, $N_{y}$ are the numbers of grids in the x and y directions, respectively.

**Figure 4:**
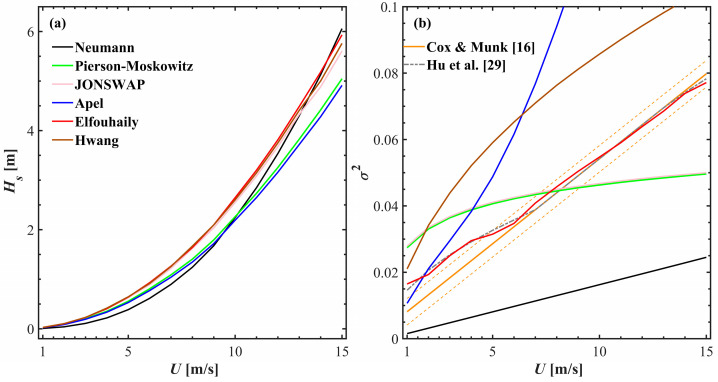
Property statistic from simulated sea surface topography based on finite differences: (**a**) significant wave height, $H_{s}$, (**b**) total mean square slope, $\sigma^{2}$. This plot uses the same legend as used for the spectrum of Figure 1. The solid and dashed orange and dashed grey lines are reprinted from Equation (3) and Equation (4), respectively. Wind speeds range from 1 m s^−1^ to 15 m s^−1^.

The significant wave heights and total MSS are calculated for each state via Equations (11) and (12). Figure 4 shows the statistical distributions of $H_{s}$ and $\sigma^{2}$ across various types of sea surfaces for wind speeds ranging from 1 m s^−1^ to 15 m s^−1^. Figure 4a depicts the increase in $H_{s}$ across different spatial surfaces as the wind speed increases. The variations among different spectra are slight, as also noted in Figure 3. In contrast, in Figure 4b, clear differences are apparent in the MSS corresponding to different sea spectra. By employing the statistical models from [16,29] as benchmarks, the Elfouhaily spectrum yields the most statistically accurate results. The refined Elfouhaily spectrum aligns closely with the Hu et al. model at low wind speeds (1 m s^−1^ to 5 m s^−1^). The Neumann spectrum consistently yields the lowest results across all wind speed conditions, whereas the Pierson–Moskowitz spectrum and the JONSWAP spectrum show alignment, corroborating Equation (8) and Figure 1f–h. The larger results of the Hwang spectrum and the Apel spectrum compound the phenomenon of high values of their slope spectra in the range of 10^2^ rad m^−1^ to 10^3^ rad m^−1^, especially with a secondary spectral peak of the Apel spectrum near 750 rad m^−1^.

## 3. Underwater–Air Channel Model

Given the high cost and complexity of field experiments, the channel modeling and performance evaluation of laser transmission links are considered vital preparatory steps before constructing an operational system [10,11,12,20]. On the basis of the photon weighting method, this study employs the ray tracing technique and Monte Carlo simulations to assess the energy prediction of laser rays within channels.

This simulation process focuses on determining the ray paths through three key channels: the seawater channel, the sea surface, and the air channel. Each path calculation relies on the probability distributions associated with these media, which include factors such as the minimum path length of the laser rays, the scattering direction, and the optical properties of each medium. Finally, the properties of the laser beam at the receiving plane are counted to gather information about the light field. To highlight the impact of the complex sea surface on laser uplink transmission, several aspects of the seawater and air channels are simplified, and the background radiation is ignored.

### 3.1. Seawater and Air Channel

The simulation presumes a homogeneous seawater column. The primary factors influencing the seawater channel are the absorption coefficient, a, and the scattering coefficient, b [45]. In this study, a chlorophyll concentration of 0.5 mg m^−3^ is selected to be the counterpart of the JII classification (a = 0.0523 m^−1^, b = 0.255 m^−1^), which can effectively encompass a broad range of open ocean environments [46,47].

The Monte Carlo simulation approach underwater can follow the process (publicly available from Refs. [17,22]): at each geometric transmission path *r*, where $r=-\frac{ln(\zeta )}{a+b}$, and ζ is a random number between 0 and 1. Then, the weight and direction of ray propagation are determined based on Inherent Optical Properties (IOPs):

(13) $$w_{i+1}=w_{i}\cdot \frac{b}{a+b}$$

(14) $$\tilde{\beta }\left(\vec{\xi_{i+1}}\to \vec{\xi_{i}}\right)d\Omega (\vec{\xi })=\tilde{\beta }(\theta_{i},\varphi_{i})\sin\theta_{i}d\theta d\varphi$$

where $w_{i}$, $\vec{\xi_{i}}$ and $w_{i+1}$, $\vec{\xi_{i+1}}$ are the ray weights and directions before and after traversing a transmission path, respectively.$\left(\theta_{i},\varphi_{i}\right)$ represent the scattering angles of the incident ray of $\vec{\xi_{i}}$ in a coordinate system, and $\tilde{\beta }$ is the scattering phase function (SPF).

The primary mechanism by which the air channel influences laser transmission is similar to that of seawater. This study concentrates solely on the impact of aerosol components, deliberately excluding factors such as clouds and turbulence, thereby establishing a cloudless standard atmosphere model [48]. Table 3 presents the optical thicknesses of aerosol stratifications with a visibility of 20 km. The scattering phase function for these layers is calculated via Mie scattering theory.

### 3.2. Sea Surface Channel

In Section 2, two sea surface models, statistical and spatial, are introduced. To compute the interaction of laser rays with the sea surface, the wave front normal vector $\vec{n}$ of the surface must first be determined. In the statistical model, the vector is derived from the PDF of sea surface slopes and the assumption of isotropy in the direction. In contrast, regarding the spatial model, we employ a spatial discretization approach, in which each rectangular grid cell is subdivided into two triangular facets—one in the upper half and one in the lower half. Given the coordinates of the three vertices for each triangle, the corresponding normal vector is computed using the cross product.

Tracing ray paths in three-dimensional space over a sea surface is akin to determining the intersection of a line with a plane [25]. The laser rays are transmitted from seawater to air, where the refractive index of water is $n_{1}=$ 1.34 and that of air is $n_{2}=$ 1. The incidence vector $\vec{I}$ and the wave front normal vector $\vec{n}$ are unit vectors, meaning that $\left|\vec{I}\right|=\left|\vec{n}\right|=$ 1. By applying Snell’s law directly with the vectors, the transmission vector $\vec{T}$ for the laser ray can be derived as follows [49]:

(15) $$\vec{T}=n_{1}\;\vec{I}-c\;\vec{n}$$

where c can be considered constant and is calculated from

(16) $$c=n_{1}\;\vec{I}\cdot \vec{n}-\sqrt{n_{1}\;{\left(\vec{I}\cdot \vec{n}\right)}^{2}-n_{1}^{2}+1}.$$

Before Equation (15), it is necessary to verify that the ray impinges on the exact wave facet and that the vector direction always transmits upward (defined as the z-axis positive) [17]. When the angle of incidence is greater than the critical angle, ~48.3°, no beam is transmitted, and total reflection occurs.

**Figure 5:**
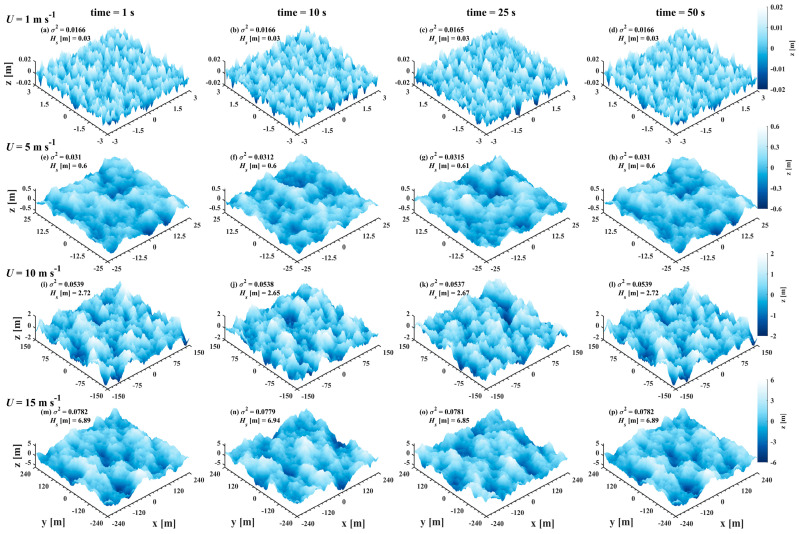
Spatial surface topographies with temporal variations for wind speeds of (top to bottom) 1, 5, 10 and 15 m s^−1^ and moments of (left to right) 1, 10, 25, and 50 s. Each spatial sea surface scale contains at least one wave wavelength.

The spatial sea surface with temporal characteristics is further simulated, and the wave spectrum is the Elfouhaily spectrum. The time interval is 1 s, totaling 50 sea surface topographies per wind speed. Figure 5 displays spatial surfaces for a time series. The waves propagate toward the x-axis positively as time progresses, and their statistical properties remain consistent. The average MSS of the spatial model is shown in Table 4, and the results are closer to those of the Hu model than to those of the Cox–Munk model, especially at 1 m s^−1^. Therefore, in this study, the statistical model utilizes a combination of Equations (1) and (4).

The parameters for the cross-media uplink laser transmission channel in the simulation are presented in Table 5. The laser operates at 532 nm, and the telescope parameters are assumed to be optimized at the receiver plane. To increase the accuracy of the Monte Carlo simulations, each simulation is conducted with more than 10^9^ photon packets.

## 4. Results and Discussion

**Figure 6:**
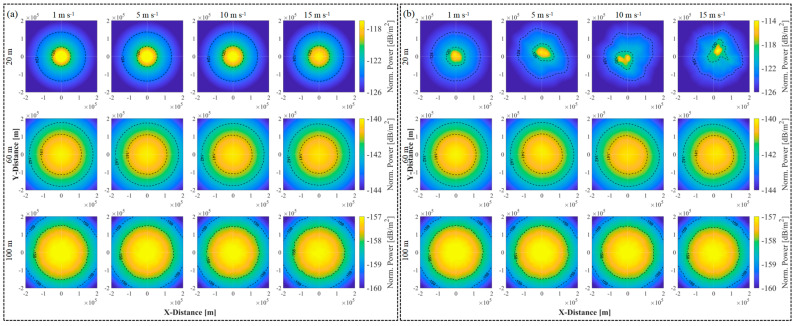
Spatial distribution of beam power in the receiving plane. The sea surface channel is (**a**) statistical model, (**b**) spatial model (t = 2 s) based on the Elfouhaily spectrum, for wind speeds of (left to right) 1, 5, 10 and 15 m s^−1^ and underwater transmission depths of (top to bottom) 20, 60 and 100 m, respectively.

To assess the impact of the sea surface on laser transmission, the beam power distributions through an underwater air channel are first simulated. Figure 6 shows the beam power distributions across a 200 × 200 km^2^ area of the receiving plane, following the application of the statistical and spatial models. In the statistical model (Figure 6a), the peak power decreases as the underwater transmission depth and the wind speed increase, whereas the shape essentially remains invariable. The performance of a laser beam is influenced primarily by the seawater link rather than by the wind speed. In contrast, the spatial model prominently exhibits variations characterized by fluctuations. Figure 6b first shows that after 20 m of underwater transmission, the high-intensity beam narrows and the maximum power at the center is slightly lower than that predicted by the statistical model. Moreover, the beam shape becomes distorted, deviating from the expected Gaussian profile. This distortion decreases as the underwater transmission depth increases.

**Figure 7:**
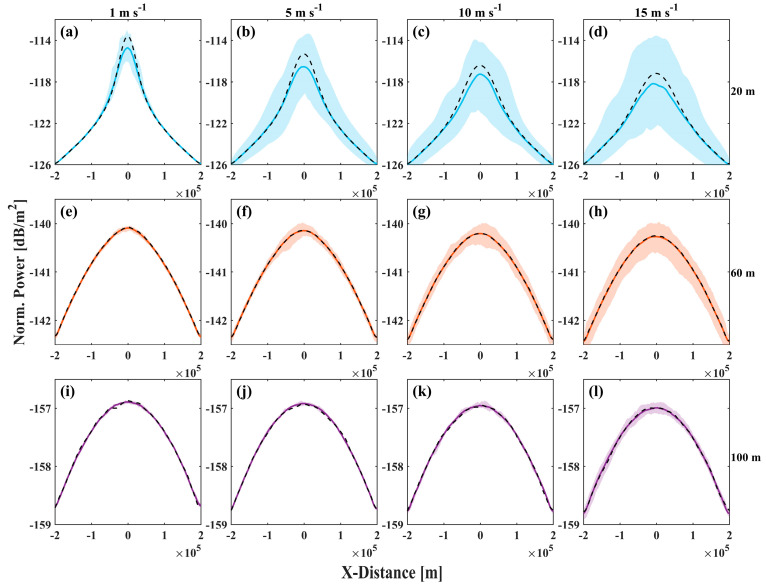
(**a**–**l**) Cross-sectional power distribution along the x-axis for wind speeds of (left to right) 1, 5, 10 and 15 m s^−1^ and underwater transmission depths of (top to bottom) 20, 60 and 100 m, respectively. The shaded area shows the statistics after 50 simulated spatial sea surface topography transmissions with the mean power (solid line) in simulations. And the black dashed line is the result of a statistical sea surface, corresponding to Figure 6a.

The sea surface in a real ocean environment is also subject to temporal variations, as illustrated in Figure 5. These changes over time may induce different distortions and drifts of the laser beam. Figure 7 presents the statistical variation in the normalized received power along the x-axis over a consistent time interval. At a transmission depth of 20 m underwater, different wind speeds influence the distortion and deviation of the laser beam differently. Concurrently, the average normalized power peak value is reduced by approximately 0.9 dB under the statistical model at similar conditions. With increasing underwater transmission depth (60 m, 100 m), the beam power distributions and shapes predicted by the spatial model align more closely with those of the statistical model, and the spatiotemporal characteristics of the sea surface exert a diminishing influence on the beam. This finding indicates that extending the optical path length in turbid waters may mitigate the scintillation effects attributed to the sea surface, although it necessitates precise adjustment of the beam power and transmission depth to optimize performance [4,15,18].

**Figure 8:**
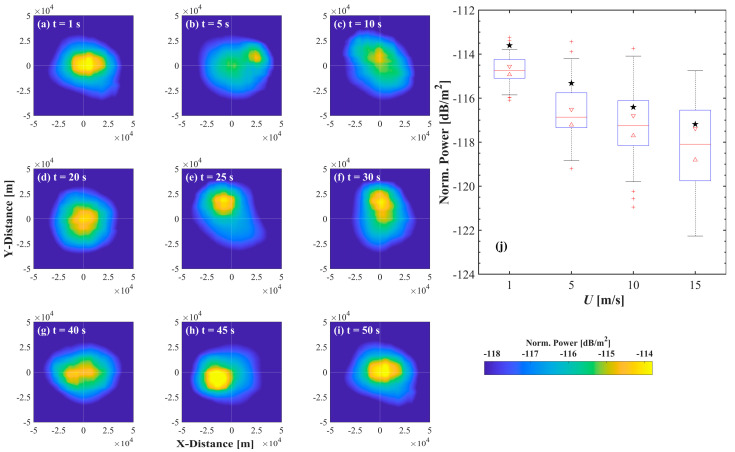
(**a**–**i**) Examples of spatial distribution of beam power across simulated sea surface at various moments for a wind speed of 1 m s^−1^ and an underwater transmission depth of 20 m. (**j**) Boxplot of power error at (x=0, y=0), corresponding to Figure 7a–d, for wind speeds of 1, 5, 10 and 15 m s^−1^ and an underwater transmission depth of 20 m. And stars indicate the results from the statistical model.

To further elucidate the preliminary results presented in Figure 7a–d, Figure 8a–i depict the dynamics of a high-intensity laser beam transmitted 20 m underwater and interacting with sea surfaces under a wind speed of 1 m s^−1^ at different times. The results demonstrate a clear relationship between the spatial distribution of beam power and the temporal and spatial characteristics of the sea surface. Specifically, the beam shapes illustrated in Figure 8a,i exhibit similarities, corresponding closely to the comparable sea surface topographies at 1 s and 50 s in Figure 5. And Figure 8j shows that the average power at the center of the space (x = 0*,* y = 0) gradually decreases as the wind speed increases. The fluctuations in the sea surface primarily influence the variability in link power [2]. However, owing to the considerable drift caused by spatial sea surfaces, the average power of the spatial model may always be lower than that of the statistical model.

Owing to the distortion effects induced by the spatial model, a suitable positioning method must be adopted to enhance the energy features and anti-interference capabilities of the spot center. This study uses the square-weighted centroid positioning method to quantify the deviation of the laser spot [50]. The centroid ($x_{c}$, $y_{c}$) can be expressed as

(17) $$\begin{array}{c} x_{c}=\frac{\sum_{i,j}^{N_{i},N_{j}}\;{{\;w}_{i,j}}^{2}\;x_{i,j\;}\;}{\sum_{i,j}^{N_{i},N_{j}}\;{{\;w}_{i,j}}^{2}} \end{array}$$

and

(18) $$\begin{array}{c} y_{c}=\frac{\sum_{i,j}^{N_{i},N_{j}}\;{w_{i,j}}^{2}y_{i,j}}{\sum_{i,j}^{N_{i},N_{j}}\;{w_{i,j}}^{2}} \end{array}$$

where $w_{i}$ is the energy weight at the coordinate ($x_{i,j}$, $y_{i,j}$).

**Figure 9:**
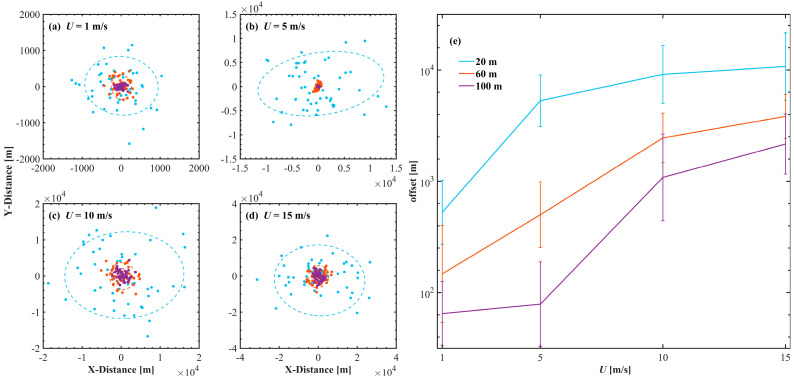
The centroid distribution of beam power on the receiving surface (**a**–**d**) corresponds to wind speeds of 1, 5, 10 and 15 m s^−1^, and the centroid offset distance (**e**). The color indicates the depth of underwater transmission. This plot uses the same legend as Figure 7.

**Figure 10:**
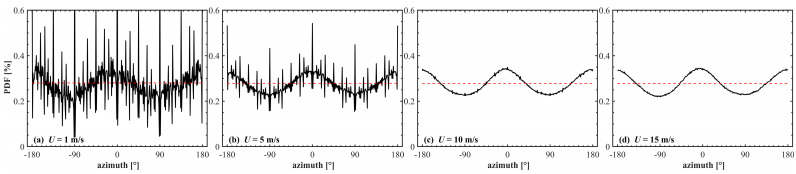
Statistical azimuth angles of simulated sea surfaces for wind speed of (left to right) 1, 5, 10 and 15 m s^−1^. The black line represents the spatial sea surface calculated from finite differences and the red line indicates the isotropic distribution of the statistical sea surface.

Figure 9 shows the spatial distribution of the centroid and the offset distance calculated in this simulation experiment. As the wind speed increases, the centroid of the spot shifts further from the initial emission position. Furthermore, enhancing the underwater transmission depth can decrease the extent of laser deviation, as corroborated by the results in Figure 7. Notably, as depicted in Figure 9a–d, the distribution of the centroid of the energy of the laser spot in space presents an elliptical structure rather than an ideal circular symmetric structure. To analyze this phenomenon, the azimuth directional statistics of the two sea surface models are separately computed and depicted in Figure 10.

Due to the simulation parameter settings, the spatial model resolutions are higher at low wind speeds, resulting in sharp jittering of the simulated sea surface at 1 and 5 m s^−1^ compared to that at 10 and 15 m s^−1^. And the high wind speeds mainly reflect the statistical distribution trend of the sea surface angle. The spatial model exhibits an anisotropic *W*-shaped distribution of the azimuth, aligning with the fundamental sea surface slope model and capturing the directional variability [16,51]. The statistical model, on the other hand, assumes an isotropic sea surface distribution. The *W*-shaped distribution of sea surface directions effectively corresponds to the elliptical distribution, highlighting the directional characteristics of the sea surface. Additionally, as wind speed and underwater transmission depth increase, the coordinate distribution gradually approaches isotropy, aligning with the assumptions of statistical modeling [52].

At low wind speeds, this study improves the performance of MSS estimation by refining truncation errors in the Elfouhaily spectrum. This enhancement is particularly important for optical applications, where surface roughness plays a critical role in beam scattering and reflection properties. As a result, the simulated sea surface demonstrates strong agreement with the existing empirical models and remote sensing observations under typical ocean conditions, making it suitable for simulating a wide range of realistic virtual scenarios.

These simulation results, which focus on the spatial and temporal dynamics of the sea surface, provide valuable insights into how these factors affect signal propagation. The sea surface characteristics influence important communication metrics, such as signal attenuation, scattering, and multipath fading, which directly impact signal-to-noise ratio (SNR), bit error rate (BER), and data throughput. For example, under varying transmission depths and wind speeds, the average beam energy in space fluctuates due to the time evolution of the dynamic sea surface. These fluctuations result in significant variations in the received energy, which subsequently affects the SNR and alters the BER of the communication performance. Additionally, the random drift of the laser across the dynamic sea surface impacts the receiver’s ability to track and scan the collimated signal. Given the sensitive nature of laser communications, this can lead to the loss of the received signal.

Therefore, developing a comprehensive reference model for evaluating optical transmissions in challenging marine environments is both essential and fundamental for improving the accuracy and reliability of WOC systems.

## 5. Conclusions

In this study, we simulate uplink laser transmission through a seawater–sea surface–air channel via ray tracing technology and the Monte Carlo simulation method, with a focus on the impact of the sea surface channel. Owing to the widely used classical statistical sea surface model, a spatial sea surface model with temporal and spatial characteristics can be implemented on the basis of a wave spectrum and FFT technology. We assessed the validity and applicability of six typical wind wave spectra, concentrating on their ability to characterize the optical properties of the sea surface, particularly the MSS. Additionally, we refined the truncation of the Elfouhaily spectrum under low-wind-speed conditions, ensuring that its variance spectrum and slope spectrum remain valid across all wavenumbers and wind speeds. By comparing different MSS models and simulated virtual sea surface states, we find that the Elfouhaily spectrum can most effectively characterize the optical sea surface and align closely with the Hu model, which was derived from actual laser data.

We applied both sea surface models for an uplink simulation experiment. The classical statistical model lacks information on spatial and temporal variations, whereas the spatial model can capture power fluctuations driven by dynamic changes in the sea surface. The simulation results indicate that the receiving power through both models diminishes with increasing wind speed. Nonetheless, deeper underwater transmission can compensate for the beam distortion and drift caused by the sea surface channel.

Importantly, deviations between the spatial model and commonly employed statistical models are still considerable. Specifically, at shorter underwater transmission distances, the spatial model may induce considerable drift in the laser output, potentially resulting in lower average power evaluations than those of the statistical model. Furthermore, the azimuth angle of the sea surface follows a W-shaped pattern rather than an isotropic distribution. This behavior results in an elliptical configuration of the energy centroid distribution rather than the expected circular shape.

Grasping the spatiotemporal correlation within the sea surface channel is essential for analyzing the laser transmission characteristics at the water–air interface. These insights can help optimize cross-media laser links, such as forward transfer modeling, LiDAR simulations and beam tracking, and improve the accuracy and reliability of systems operating in complex marine environments.

## Figures and Tables

**Table 1:** Several typical candidate wave spectra.

Spectrum	Input Parameter	Description
Neumann [33]	wind speed	Growth of wind-generated ocean waves
Pierson-Moskowitz [34]	wind speed	Steady wind & fully developed sea state model
JONSWAP [35]	parameters of fetch	Enhanced peakness & rapid energy growth under wind
Apel [36]	wind speed & wave age	Model for Radar applications
Elfouhaily [37]	wind speed & wave age	Unifies low and high wavenumbers for optical data
Hwang [38]	wind speed, wave age & influence parameters	Model from tropical cyclones

**Table 2:** Parameters of the simulated sea surface.

**Parameters**	**U [m s^−1^]**			
	**1**	**5**	**10**	**15**
L [m]	6	50	300	480
N	240	2000	3000	2400

**Table 3:** Vertical distribution and optical thickness of aerosol stratifications [from Ref. [48]].

Layer [km]	Aerosol Type	τ
0~2	maritime	0.26
2~12	continental	0.05
12~50	sulfate	0.005

**Table 4:** Comparison of sea surface mean square slopes across different models.

Model		$\sigma^{2}$[×10^−3^] at Wind Speed [m s^−1^]			
		1	5	10	15
Cox & Munk [16]		8.12	28.6	54.2	79.8
Hu et al. [29]		14.6	32.6	54.2	78.3
Elfouhaily Spectrum	origin	6.89	31.5	53.9	78.1
	Refined	16.6			

**Table 5:** Parameters for seawater–air laser uplink transmission channel.

Setting		Parameter	Value
Initial laser		Shape	Gaussian
		Wavelength [nm]	532
		Beam waist [mm]	2
		Divergence angle [mrad]	2
Receiver plane		Telescope diameter	Optimal
		Field of view [rad]	π
		Height [km]	500
Channel	Seawater	Type	JII & homogeneous
		Depth [m]	20, 60 & 100
	Sea surface	Model	Statistical model (Hu model)
			Spatial model (Elfouhaily spectrum)
		Wind speed [m s^−1^]	1, 5, 10, 15
	Air	Preliminary cloudless standard atmosphere (see in Table 3)	

## Data Availability

The inputs and outputs of model simulation in this study are available upon request to the corresponding author. The method for simulating sea surfaces used in this study is publicly available from Refs [24,43].

## References

[1] Luo H., Wang J., Bu F., Ruby R., Wu K., Guo Z. (2022). Recent Progress of Air/Water Cross-Boundary Communications for Underwater Sensor Networks: A Review. IEEE Sens. J..

[2] Zhang F., Luo J., Li J., Lin T., Gong C., Xu Z. (2023). Effects of underwater swing nodes on water-to-air visible light communication. Appl. Opt..

[3] Dong L., Li N., Xie X., Bao C., Li X., Li D. (2020). A Fast Analysis Method for Blue-Green Laser Transmission through the Sea Surface. Sensors.

[4] Zhou T., He Y., Zhu X., Chen W.B. Influence of sea-air interface on upward laser beam propagation. Proceedings of the International Symposium on Photoelectronic Detection and Imaging 2013: Laser Communication Technologies and Systems, Proc. SPIE 8906.

[5] Di Y., Shao Y., Chen L. (2021). Real-Time Wave Mitigation for Water-Air OWC Systems Via Beam Tracking. IEEE Photonic Tech. Lett..

[6] Xu A., Di Y., Yue X., Chen L.-K. (2024). Beam Tracking aided by Complexity-reduced MobileNetV2 for Water-air OWC with Waves. IEEE Photonic Tech. Lett..

[7] Zhou Y., Chen W., Cui X., Malinka A., Liu Q., Han B., Wang X., Zhuo W., Che H., Song Q. (2019). Validation of the Analytical Model of Oceanic Lidar Returns: Comparisons with Monte Carlo Simulations and Experimental Results. Remote Sens..

[8] Huang N.E., Long S.R., Bliven L.F., Tung C.-C. (1984). The Non-Gaussian joint probability density function of slope and elevation for a nonlinear gravity wave field. J. Geophys. Res. Oceans..

[9] Chowdhary J., Zhai P., Boss E., Dierssen H., Frouin R., Ibrahim A., Lee Z., Remer L.A., Twardowski M., Xu F. (2019). Modeling atmosphere-ocean radiative transfer: A PACE mission perspective. Front. Earth Sci..

[10] Chen L., Shao Y., Di Y. (2020). Underwater and Water-Air Optical Wireless Communication. IEEE J. Light. Technol..

[11] Angara B.R., Shanmugam P., Ramachandran H. (2024). Influence of sea surface waves and bubbles on the performance of underwater-to-air optical wireless communication system. Opt. Laser Technol..

[12] Karp S. (1976). Optical Communications Between Underwater and Above Surface (Satellite) Terminals. IEEE Trans. Commun..

[13] Sun X., Kong M., Alkhazragi O., Telegenov K., Ouhssain M., Sait M., Guo Y., Jones B.H., Shamma J.S., Ng T.K. (2020). Field Demonstrations of Wide-Beam Optical Communications Through Water–Air Interface. IEEE Access.

[14] Alharbi O., Kane T., Henderson D. (2022). Impact of a Turbulent Ocean Surface on Laser Beam Propagation. Sensors.

[15] Hu Q., Gong C., Lin T., Luo J., Xu Z. (2022). Secrecy Performance Analysis for Water-to-Air Visible Light Communication. IEEE J. Light. Technol..

[16] Cox C., Munk W. (1954). Measurement of the roughness of the sea surface from photographs of the Sun’s glitter. J. Opt. Soc. Am..

[17] Leathers R.A., Downes T.V., Davis C.O., Mobley C.D. (2004). Monte Carlo Radiative Transfer Simulations for Ocean Optics: A Practical Guide.

[18] Dong Y., Tang S., Zhang X. (2013). Effect of Random Sea Surface on Downlink Underwater Wireless Optical Communications. IEEE Commun. Lett..

[19] Zhang H., Dong Y., Hui L. (2015). On Capacity of Downlink Underwater Wireless Optical MIMO Systems With Random Sea Surface. IEEE Commun. Lett..

[20] Sahoo R., Sahu S.K., Shanmugam P. (2019). Estimation of the channel characteristics of a vertically downward optical wireless communication link in realistic oceanic waters. Opt. Laser Technol..

[21] Ata Y., Kiasaleh K. (2024). Performance of optical seawater-to-air wireless links in the presence of seawater pitching angle effect. IEEE Trans. Commun..

[22] Li C., Yuan R., Gao H., Zhang T., Sun B., Chen T., Cao G. (2021). Characteristics of Blue-green Laser Downlink Cross-media Transmission under Different Weather Conditions. Acta Photonica Sinica.

[23] Jiao C., He Y., Hu S., Liu H., Chen W., Zhang W. (2024). Effects of Solar Radiation on the Performance of Long-distance Atmosphere-ocean Laser Communication Links. Opt. Commun..

[24] Mobley C.D. (2015). Polarized reflectance and transmittance properties of windblown sea surfaces. Appl. Opt..

[25] Zhou T., Chen W., He Y., Zhu X. (2010). Beam Spatial Distribution of Upward Laser Through Sea-Air Interface. Chin. J. Lasers..

[26] Preisendorfer R.W. (1976). Hydrologic Optics: Volume VI Surfaces.

[27] He M., Hu Y., Huang J., Stamnes K. (2016). Aerosol optical depth under “clear” sky conditions derived from sea surface reflection of lidar signals. Opt. Express.

[28] Tang Q., Hu Y., Li W., Huang J., Stamnes K. (2018). Optimizing cirrus optical depth retrievals over the ocean from collocated CALIPSO and AMSR-E observations. Appl. Opt..

[29] Hu Y., Stamnes K., Vaughan M., Pelon J., Weimer C., Wu D., Cisewski M., Sun W., Yang P., Lin B. (2008). Sea surface wind speed estimation from space-based LIDAR measurements. Atmo. Chem. Phys..

[30] McAllister M.L., Draycott S., Calvert R., Davey T., Dias F., Bremer T.S.v.D. (2024). Three-dimensional wave breaking. Nature.

[31] Jhunjhunwala A., Rao M.M., Rajendran V., Rama S.K., Jeoti V. (1986). Effect of Ocean Surface on Laser Communication Link from Ground to Submarine. IETE J. Res..

[32] Lynch D.K., Dearborn DS P., Lock J.A. (2011). Glitter and glints on water. Appl. Opt..

[33] Neumann G. (1952). On Wind Generated Ocean Waves with Special Reference to the Problem of Wave Forecasting.

[34] Pierson W.J., Moskowitz L. (1964). A proposed spectral form for fully developed wind seas based on the similarity theory of SA Kitaigorodskii. J. Geophys. Res..

[35] Hasselmann K., Barnett T.P., Bouws E., Carlson H., Cartwright D.E., Enke K., Ewing J.A., Gienapp A., Hasselmann D.E., Kruseman P. (1973). Measurements of Wind-Wave Growth and Swell Decay during the Joint North Sea Wave Project (JONSWAP).

[36] Apel J.R. (1994). An improved model of the ocean surface wave vector spectrum and its effects on radar backscatter. J. Geophys. Res..

[37] Elfouhaily T., Chapron B., Katsaros K., Vandemark D. (1997). A unified directional spectrum for long and short wind-driven waves. J. Geophys. Res..

[38] Hwang P.A., Fan Y., Ocampo-Torres F.J., García-Nava H. (2017). Ocean Surface Wave Spectra inside Tropical Cyclones. J. Phys. Oceanogr..

[39] Alves J.-H., Banner M.L., Young I.R. (2003). Revisiting the Pierson–Moskowitz Asymptotic Limits for Fully Developed Wind Waves. J. Phys. Oceanogr..

[40] Mitsuyasu H. (2002). A Historical Note on the Study of Ocean Surface Waves. J. Oceanogr..

[41] Hara T., Bock E.J., Lyzenga D. (1994). In situ measurements of capillary-gravity wave spectra using a scanning laser slope gauge and microwave radars. J. Geophys. Res..

[42] Jähne B., Riemer K.S. (1990). Two-Dimensional Wave Number Spectra of Small-Scale Water Surface Waves. J. Geophys. Res..

[43] Tessendorf J. (2004). Simulating Ocean Water.

[44] Kay S., Hedley J.D., Lavender S., Nimmo-Smith A. (2011). Light transfer at the ocean surface modeled using high resolution sea surface realizations. Opt. Express.

[45] Cox W., Muth J. (2014). Simulating channel losses in an underwater optical communication system. J. Opt. Soc. Am..

[46] Morel A., Maritorena S. (2001). Bio-optical properties of oceanic waters: A reappraisal. J. Geophys. Res..

[47] Mobley C.D. (2022). The Oceanic Optics Book.

[48] World Climate Research Programme (WCRP) (1986). A Preliminary Cloudless Standard Atmosphere for Radiation Computation.

[49] Guo K., Li Q., Mao Q., Wang C., Zhu J., Liu Y., Xu W., Zhang D., Wu A. (2021). Errors of Airborne Bathymetry LiDAR Detection Caused by Ocean Waves and Dimension-Based Laser Incidence Correction. Remote Sens..

[50] Guo Q., Zhang Y., Lin X., Yu T. (2018). Centroid locating method of spaceborne laser altimeter ground-based spot. Chin. J. Electron..

[51] Gao H., Li N., Zhang T., Romanic D., Wright J.S., Guan L. (2024). A Generalized Model of Sea Surface Slopes and Its Application to Sun Glint Correction on HY-1C/COCTS Imagery. IEEE Trans. Geosci. Remote Sens..

[52] Zhang H., Wang M. (2010). Evaluation of sun glint models using MODIS measurements. J. Quant. Spectrosc. Radiat. Transf..

